# Supplementary material for: Inner centromere localization of the CPC maintains centromere cohesion and allows mitotic checkpoint silencing
Source: Nat Commun. 2017 May 31;8:15542. doi: 10.1038/ncomms15542 (PMC5460030; doi:10.1038/ncomms15542)
Supplement: Supplementary Information — Supplementary Figures and Supplementary References [file ncomms15542-s1.pdf]

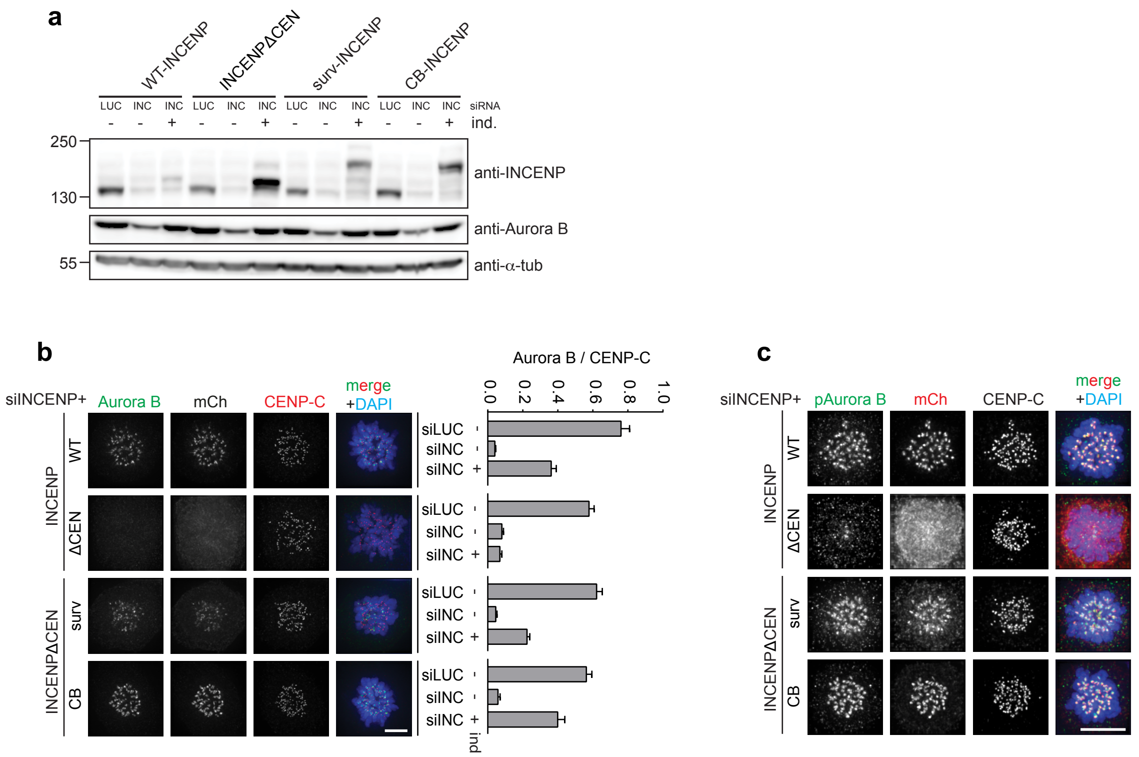


**Supplementary Figure 1 (related to Figure 1) | Expression, localization and functionality of INCENP variants used in this study.** (**a**) Western blot showing the expression levels of the indicated INCENP variants compared to endogenous INCENP in HeLa Flp-In T-REx cells -/+ induction (ind.) by doxycyclin for 8 hrs. (**b**) IF of Aurora B, mCherry and CENP-C in cells +/- induction of the indicated mCherry-tagged INCENP variants, transfected with the indicated siRNAs and blocked in mitosis with STLC. Quantification of the fluorescence intensities of Aurora B/CENP-C is shown on the right (1 exp. out of 2, 11 cells/condition, mean ± s.e.m.). (**c**) IF of phospho-Aurora B (T232, pAurora B), mCherry and CENP-C. Scale bars: 5 µm.

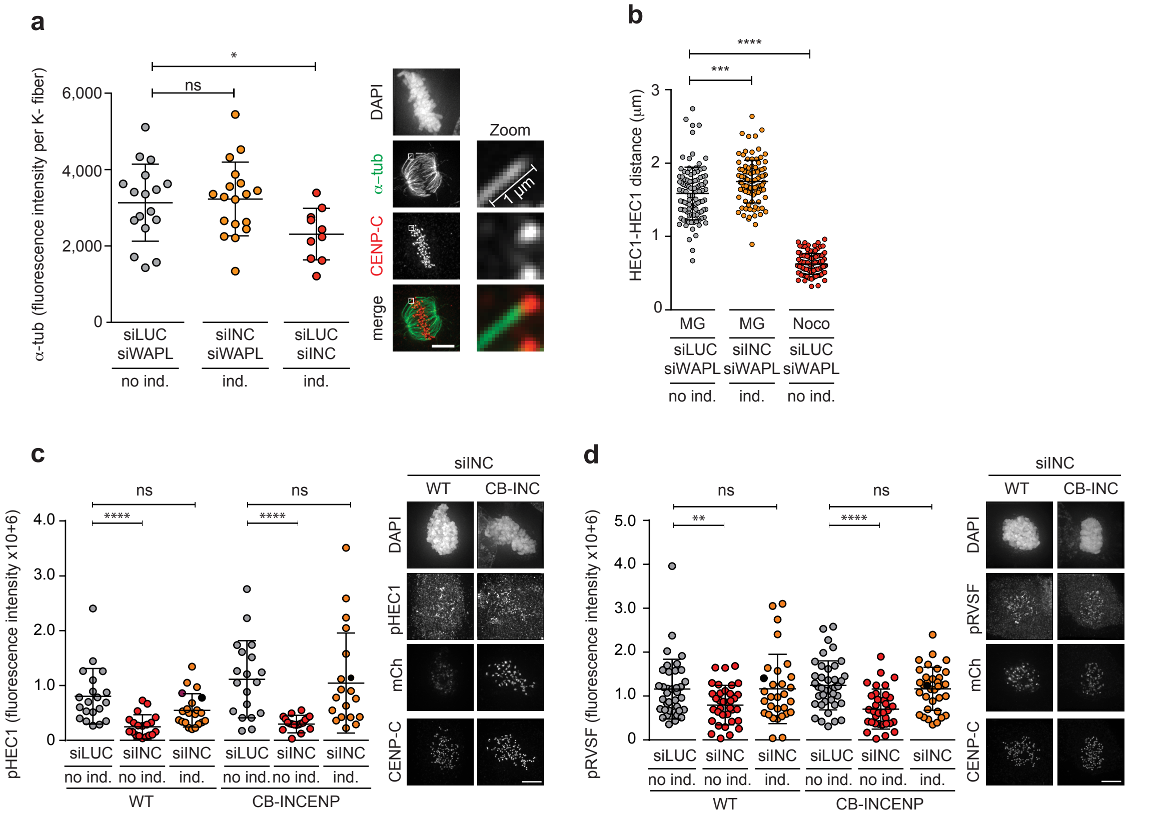


**Supplementary Figure 2 (related Figures 4 and 5) | Establishment of tension-generating, cold-stable KT-MT attachments and phosphorylation of HEC1 and KNL1 in CB-INCENP expressing cells.** (**a**) IF for α-tubulin and CENP-C in cells transfected with the indicated siRNAs and subjected to the bi-orientation assay (Fig. 1c) followed by ice-cold treatment. Quantification of α-tubulin fluorescence intensity in regions of individual K-fibers extending 1µm from the kinetochore. Example of K-fiber region selection is shown on the right (1 representative exp., 17 K fibers/condition, bars: mean ± s.d., ns=not significant; *P<0.05; unpaired t test). Scale bar: 5µm. (**b**) HEC1-HEC1 distances measured for ≥ 95 kinetochore pairs of 10 cells per condition (1 representative exp. out of 2; ***P<0.001, ****P<0.0001 ; unpaired t test). (**c, d**) Quantifications of fluorescence intensities for pHEC1, mCherry and CENP-C (**c**), or for KNL1-pRVSF, mCherry and CENP-C (**d**) (1 out of 2 exp., ≥20 cells/condition for pHEC1 and ≥35 cells for pRVSF, bars: mean ± s.d., ns=not significant; **P<0.01; ****P<0.0001; unpaired t test). The corresponding data points of the images (scale bars: 5 µm) are colored black in the graphs. DNA is visualized using DAPI.

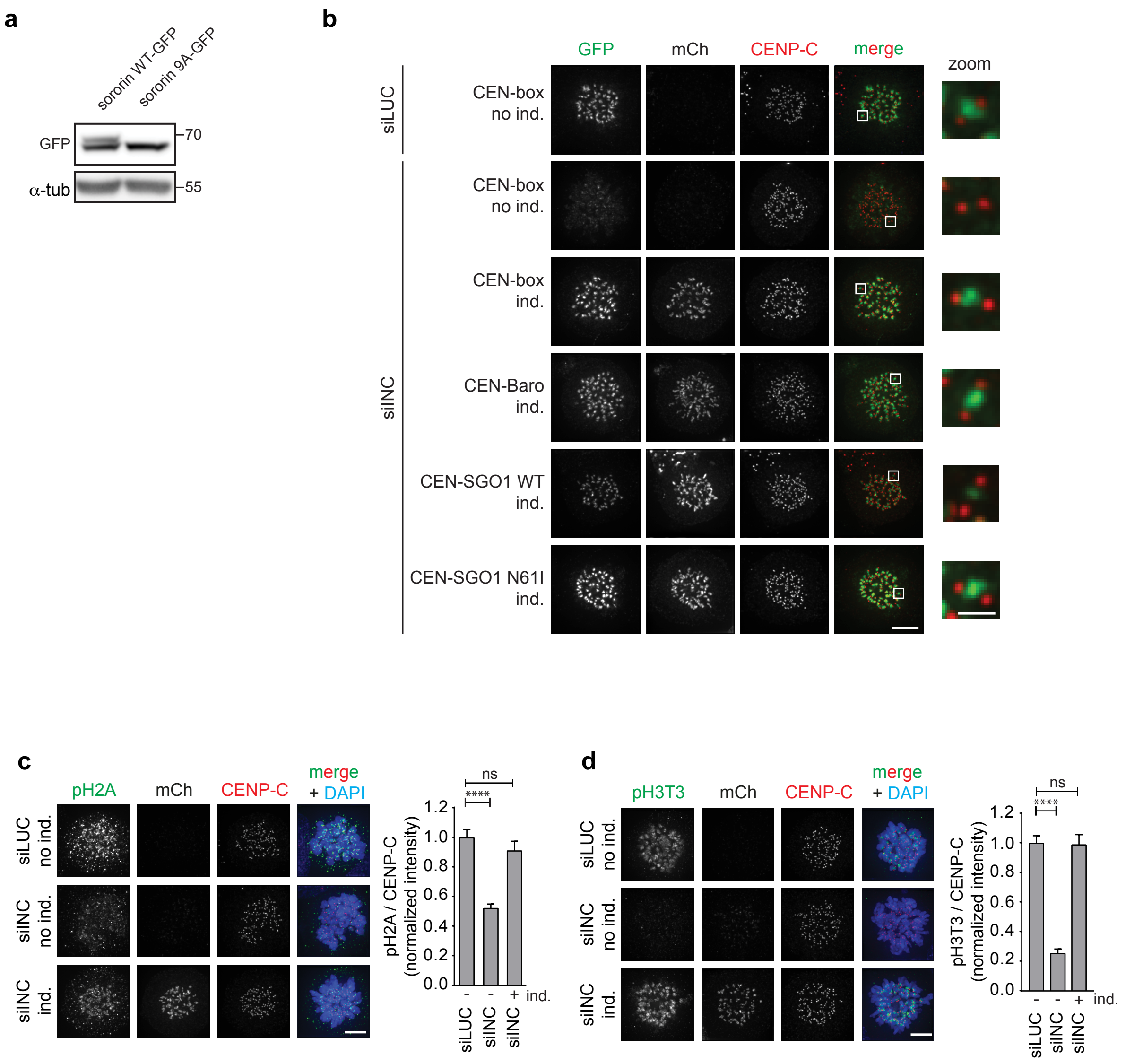


**Supplementary Figure 3 (related to Figure 4) | Expression of CB-INCENP restores phosphorylation of H3-T3 and H2A-T120 in INCENP knockdown cells which is required to localize exogenously expressed GFP-CEN-box.** (**a**) Detection of expression of sororin proteins by Western blotting after viral transduction into CB-INCENP expressing HeLa Flp-In T-REx cells. Blots were probed with anti-GFP. (**b**) IF of GFP (to visualize the GFP-tagged CEN-box, CEN-Baronase, or CEN-box SGO1 fusion proteins), mCherry and CENP-C in HeLa Flp-In T-REx cells -/+ induction CB-INCENP-mCherry and subjected to the experimental set-up as depicted in Fig. 4f-h but fixed when in STLC (Scale bar: 5µm). Enlargements of selected image regions are shown on the right (Scale bar: 1µm). (**c, d**) IF of mCherry, CENP-C and phospho-H2A-T120 (pH2A) (**c**) and phospho-H3-T3 (pH3T3) (**d**), in HeLa Flp-In T-REx cells +/- induction of CB-INCENP, transfected with the indicated siRNAs and blocked in mitosis with STLC. Quantifications of the relative fluorescence intensities (mean ± s.e.m., ns=not significant, ****P<0.0001, unpaired t test) of pH2A/CENP-C (**c**) and of pH3T3/CENP-C (**d**) are shown on the right (one representative exp. out of 3, 11 cells/condition). Scale bars: 5 µm. Of note, in contrast to Klein *et al.*[1], in our hands GFP-tagged CEN-box did not localize to the inner centromere in INCENP-depleted cells, most likely because of the lack of pH3T3 and pH2A. Phosphorylation of these histones is mediated by Haspin and BUB1[2,3], respectively, and both kinases require Aurora B activity for their activation, respectively kinetochore localization[4,5]. In the presence of endogenous INCENP or of CB-INCENP, GFP-CEN-box does localize to the inner centromere, and this correlates with the presence of the pH3T3 and pH2A.

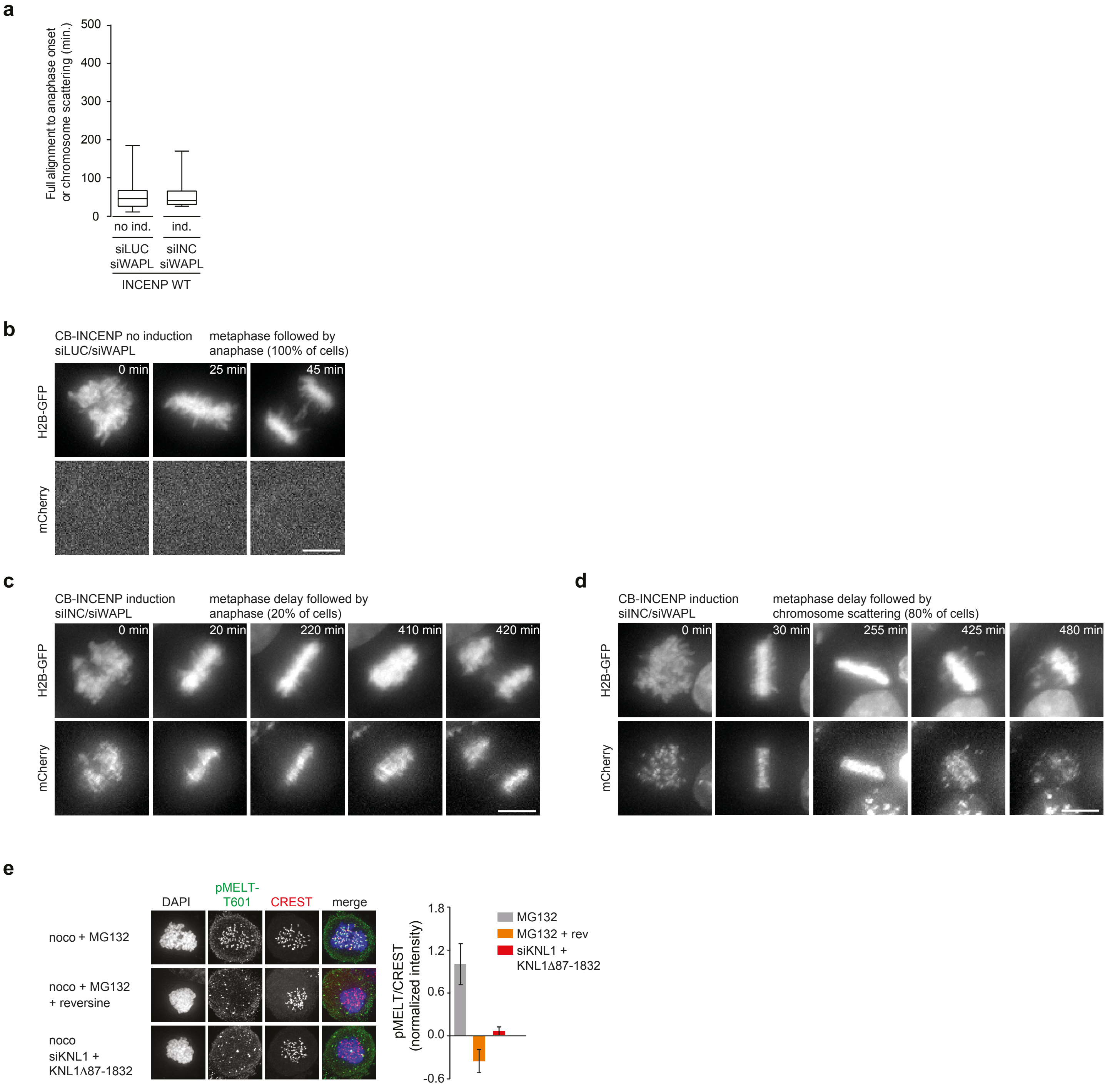


**Supplementary Figure 4 (related to Figure 5) | Metaphase delay in WAPL-depleted cells when Aurora B is localized near kinetochores.** (**a-d**) HeLa Flp-In T-REx cells stably expressing H2B-GFP and -/+ induction of WT-INCENP-mCherry (n=33 cells, **a**), or CB-INCENP-mCherry (**b-d**) were transfected with indicated siRNAs, released from a thymidine block and imaged “live” by video microscopy. (**b-d**) Stills of one representative cell for each condition and category are shown. Scale bars: 10 μm. T=0 represents the first frame after NEB, the following image represents the first frame in which all chromosomes were aligned at the metaphase plate. Time from complete alignment to anaphase or chromosome scattering was calculated and is presented in Fig. 5a. Of note, after an average 265 min. (s.d. = 128 min) delay, 20% of the CB-INCENP expressing cells went into anaphase (**b**), whilst 80% of the cells showed signs of chromosome scattering, i.e. cohesion fatigue, and remained in mitosis (**d**). This suggests that an overall increase in cohesin might not fully compensate for defective centromeric cohesion when mitotic progression is severely delayed. (**d**) Characterization of the KNL1-pMELT-T601 antibody (mean ± s.d., n=10 cells/condition). The indicated phospho-specific antibody was used to stain HeLa cells that were blocked in mitosis using nocodazole and treated with the MPS1 inhibitor reversine (plus MG132 to prevent mitotic exit). In addition, cells in which endogenous KNL1 was replaced by exogenously expressed KNL1 devoid of all TΩ-MELT-like modules (i.e. lacking aa 87-1832) was included in the analysis[6].

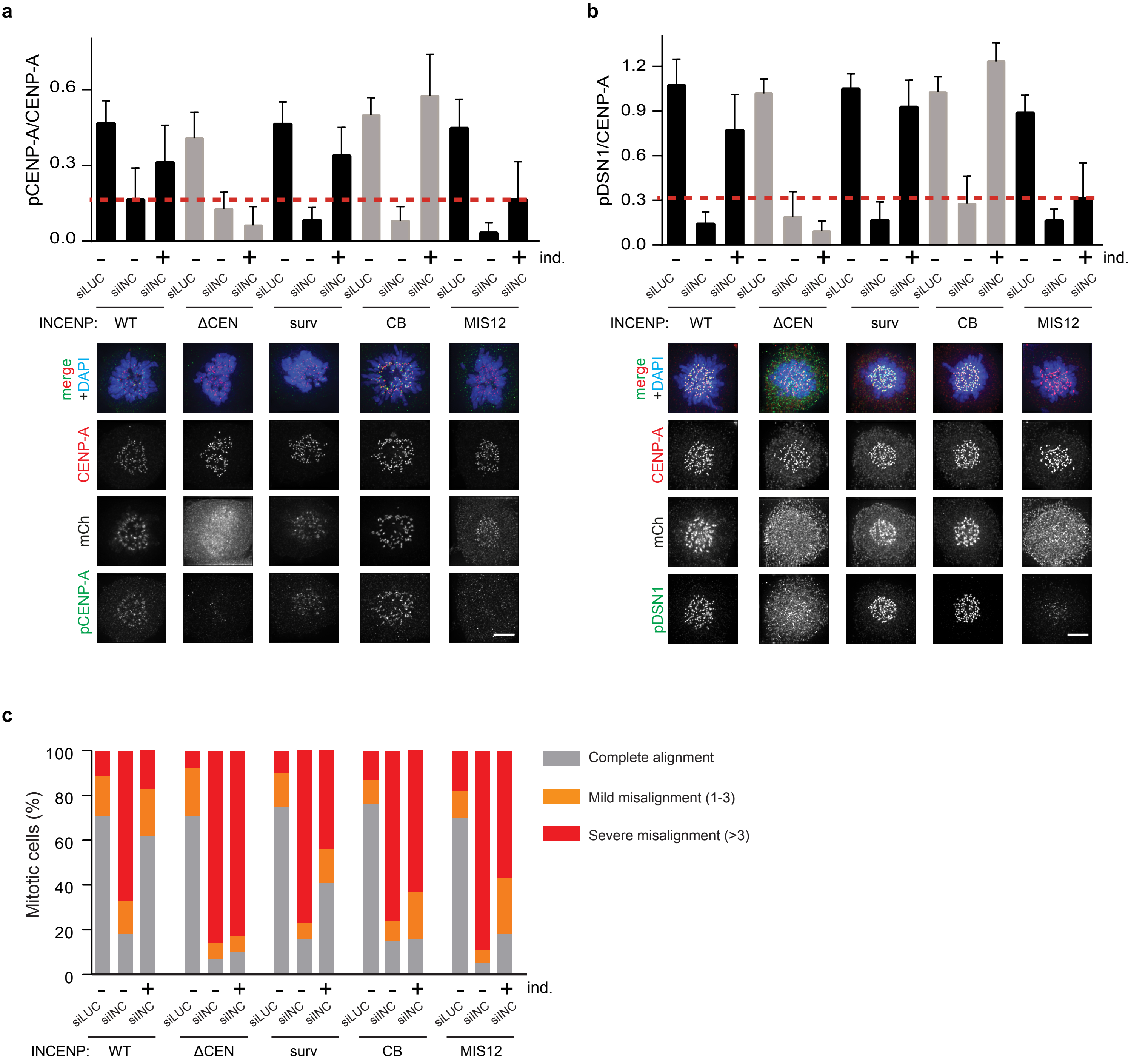


**Supplementary Figure 5 (related to Figure 5) | MIS-INCENP expression in INCENP knock-down cells.** (**a**,**b**) IF of phospho-CENP-A-S7 (pCENP-A) (**a**) or of phospho-DSN1-S109 (pDSN1)(**b**), combined with mCherry and CENP-A in HeLa Flp-In T-REx cells stably expressing the depicted mCherry-tagged INCENP variants (+ ind. = expression induced by doxycycline, - ind. = no induction of expression). Cells were transfected with the indicated siRNAs and treated with STLC. Scale bar = 5 μm, DNA is visualized using DAPI. Quantification of the fluorescence intensities of pCENP-A/CENP-A and of pDSN1/CENP-A are shown above the images (1 exp. out of 2, 15 cells/condition, mean ± s.e.m.). The red dashed line is set to the levels obtained by MIS12-INCENP. (**c**) Cells were transfected with siRNAs for Luciferase (siLUC) or INCENP (siINC) and subjected to the bi-orientation assay. Chromosome alignment was assessed (n= 80 cells/condition). The red dashed line is set to the levels obtained by MIS12-INCENP.

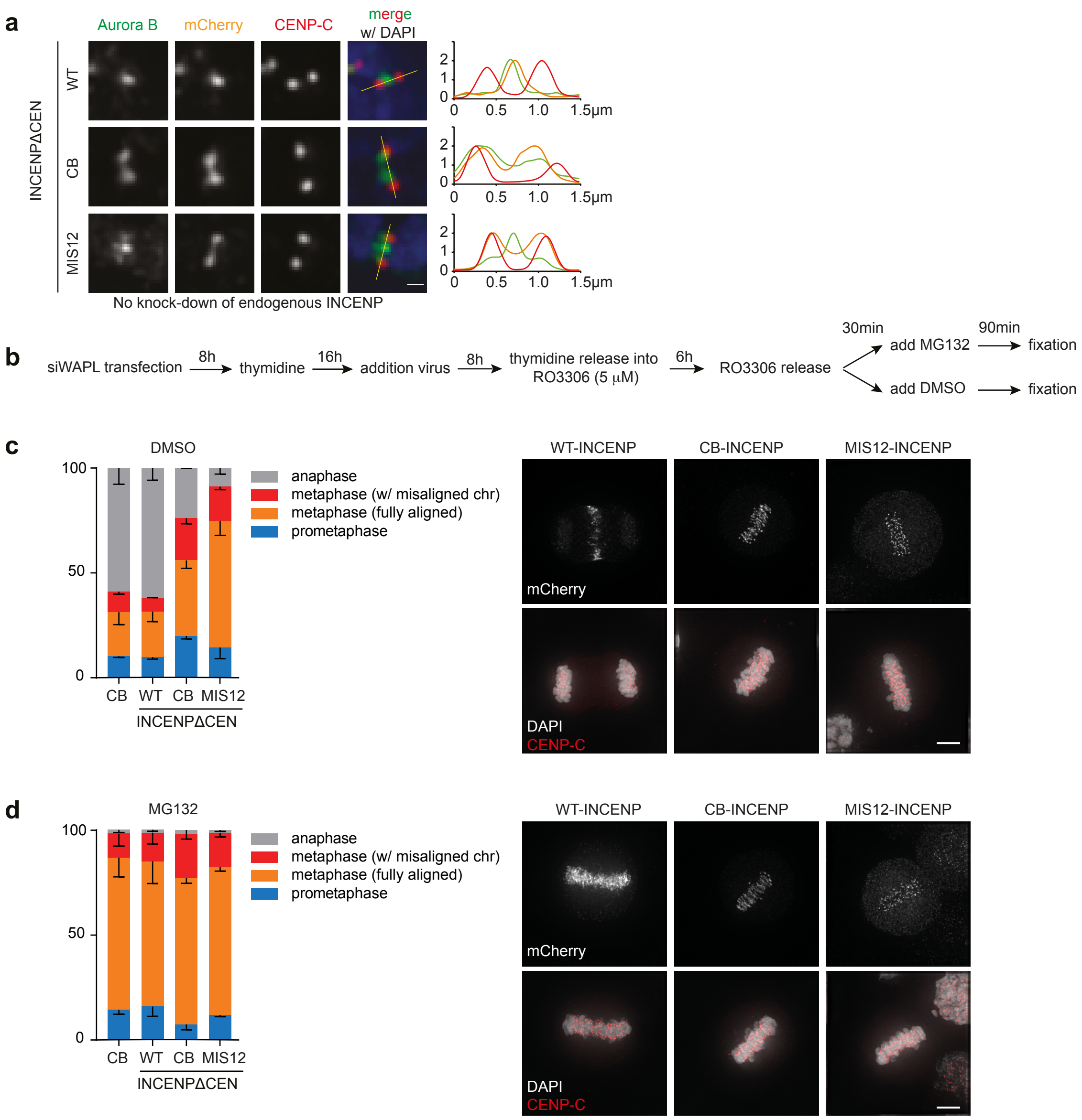


**Supplementary Figure 6 | Kinetochore localized Aurora B delays anaphase onset, but does not detach chromosomes when cohesin is stabilized.** (**a**) IF of Aurora B, RFP (to detect mCherry), and CENP-C on chromosome spreads of nocodazole treated cells transduced with WT-INCENP-mCherry, CB-INCENP-mCherry or MIS12-INCENP-mCherry expressing baculoviruses. 1D line graphs of Aurora B (green), mCherry (orange) and CENP-C (red) are shown on the right. Scale bar: 0.5 μm. Note that endogenous INCENP is present and that CB-INCENP and MIS12-INCENP have to compete with endogenous INCENP for Aurora B. Similar to previous work, CB-INCENP redistributes nearly all Aurora B away from the inner centromere towards the kinetochore, whilst MIS12-INCENP redistributes only a small amount of Aurora B onto kinetochores[7]. (**b**) Scheme of experimental set-up. (**c, d**) Cells were transfected with siWAPL and transduced with the indicated baculoviruses (according to scheme shown in b, CB-mCherry was used as control). The different mitotic phases were scored (n=2 exp., ≥200 cells/condition, error bars are s.e.m. Representative images are shown on the right (scale bar: 5μm).

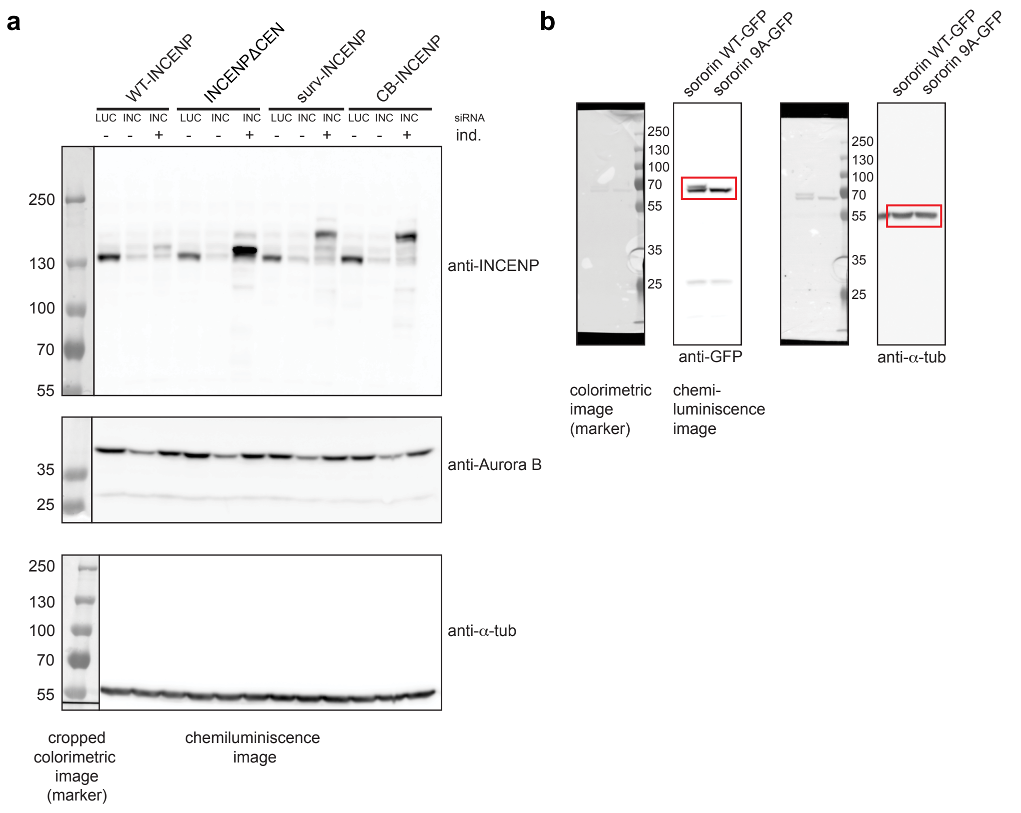


**Supplementary Figure 7 | Uncropped blots**. (**a**) Full chemiluminiscence images of cropped blots shown in Supplementary Figure 1a and (**b**) of blots shown in Supplementary Figure 3a. A colorimetric image was made of the same blot to visualize the molecular weight marker. In a) only the marker region is shown of the colorimetric image and placed on the left side of the chemiluminiscence image. In **b**) the entire colorimetric image is shown. Red square indicates the cropped area.

**Supplementary References:**


1. Klein UR, Nigg EA, Gruneberg U. Centromere targeting of the chromosomal passenger complex requires a ternary subcomplex of Borealin, Survivin, and the N-terminal domain of INCENP. *Mol Biol Cell* **17**, 2547-2558 (2006).

2. Dai J, Sultan S, Taylor SS, Higgins JM. The kinase haspin is required for mitotic histone H3 Thr 3 phosphorylation and normal metaphase chromosome alignment. *Genes Dev* **19**, 472-488 (2005).

3. Kawashima SA, Yamagishi Y, Honda T, Ishiguro K, Watanabe Y. Phosphorylation of H2A by Bub1 prevents chromosomal instability through localizing shugoshin. *Science* **327**, 172-177 (2010).

4. Wang F, Ulyanova NP, van der Waal MS, Patnaik D, Lens SM, Higgins JM. A positive feedback loop involving Haspin and Aurora B promotes CPC accumulation at centromeres in mitosis. *Curr Biol* **21**, 1061-1069 (2011).

5. Hauf S*, et al.* The small molecule Hesperadin reveals a role for Aurora B in correcting kinetochore-microtubule attachment and in maintaining the spindle assembly checkpoint. *J Cell Biol* **161**, 281-294 (2003).

6. Vleugel M*, et al.* Arrayed BUB recruitment modules in the kinetochore scaffold KNL1 promote accurate chromosome segregation. *J Cell Biol* **203**, 943-955 (2013).

7. Liu D, Vader G, Vromans MJ, Lampson MA, Lens SM. Sensing chromosome bi-orientation by spatial separation of aurora B kinase from kinetochore substrates. *Science* **323**, 1350-1353 (2009).
